# Root canal treatment of dilacerated second maxillary premolars: Planning the shaping procedure

**DOI:** 10.4317/jced.54886

**Published:** 2018-06-01

**Authors:** Davide Mancino, Naji Kharouf

**Affiliations:** 1 DDS, Université de Strasbourg, Inserm UMR-S 1121, Biomatériaux et Bioingénierie, FMTS, F-67000 Strasbourg, France; 2 DDS, Hôpitaux universitaires de Strasbourg, F-67000 Strasbourg, France

## Abstract

This article aims at investigating endodontic anatomical variants in the human maxillary premolars allowing the planning of safer and faster shaping procedures. Endodontic literature describes maxillary 2nd premolars as some of the easiest teeth to treat, as they have either one or two straight canals. Rarely they may sometimes have two dilacerated canals. This paper reports two clinical cases of maxillary premolars whose anatomy is quite unusual. In the first case report we describe a maxillary 2nd premolar with a single root and two dilacerated merged canals. The second case report concerns the retreatment of a maxillary 2nd premolar with a single root and two independent dilacerated canals, and even some lateral canal. In dilacerated anatomy, canal scouting step might cause some procedural errors. To avoid these procedural errors, we propose a modern step down technique using at first a rotary NiTi glide path instrument, to go up to the 2/3 of root canal length or until to the first impediment. This would allow an easy apical scouting of the last millimeters of endodont and increase the volume of the irrigants in the apical region. In this way since a safer and faster shaping procedure could be performed.

** Key words:**Endodontic anatomy, maxillary 2nd premolar, modern step down technique.

## Introduction

The basis for successful endodontic treatment is the clinician’s knowledge of the endodontic anatomy, its frequent variations, and individual complexities (1).

Internal anatomy of root canals is rarely as simple as the external morphology of the tooth. Human maxillary premolars are not exceptions to this rule. Endodontic literature describes maxillary 2nd premolars as some of the easiest teeth to treat, as they have either one or two straight canals (1-4).

There are exceptions to this basic anatomy. They may sometimes have two dilacerated canals. In addition, lateral ramifications of the root canal system may also occur increasing the probability of leaving untreated spaces after the root canal therapy.

We report two clinical cases describing the endodontic treatment of two dilacerated second maxillary premolar with uncommon anatomy. The first case concerns a single root maxillary 2nd premolar with two dilacerated merged canals. The second case report is about a retreatment of a single root maxillary 2nd premolar with two independents dilacerated canals, and an additional lateral canal.

It is crucial to understand how the overall canal system should be shaped, especially concerning dilacerated teeth, to avoid procedural errors (block, apical zip, canal aberrations, instrument separation) Fundamental errors can negatively influence the outcome of endodontic therapy.

## Case Reports

Case 1

A 47-year-old caucasian female was referred to the Endodontic Clinic of Dental Faculty at the University of Strasbourg). Her medical history found no outstanding findings that would contribute to treatment problems. Clinical examination revealed that the tooth had a MOD composite restoration. This was tender on percussion, and hence the patient reported periodic episodes of spontaneous pain. The periapical radiographic examination, with an orthoradial projection, showed the presence of a single root with an unusual anatomic variation, suggesting a probable endodontic dilacerated anatomy.

The endodontic treatment was performed in a single session. After local anesthesia, a rubber dam was placed, and endodontic access was performed with a # C 801L 012 round diamond bur (NTI, Kahla, Germany). The lingual and buccal canal orifices were localized with a START X 1 (DentsplySirona Ballaigues, Switzerland) using the operating microscope (Leika M320).

During all instrumentation steps an aqueous 6% NaOCl solution was used for irrigation.

In order to avoid the risk of procedural errors the strategy was was not to use initial manual scouting, but to remove immediately the coronal and middle interferences with initial rotary preflaring to then perform a manual apical scouting of last 2 mm of the root canal.

At first, an initial mechanical preflaring was performed with the OneG (Micromega Besançon, France) until just above the first root canal curvature, using an inward and outward movement, without any pressure, and then with TS1 (Micromega Besançon, France) short of 1mm in regarding to the portion of canal preflared with OneG, using an endodontic engine (300 rpm/2 Ncm).

Thanks to the initial preflaring a #10 stainless steel MMC-file (Micromega Besançon, France) scouted the canal up to working length + 0, 5 mm. Length determination was obtained using an electronic apex locator (Root ZX; J Morita Co, Kyoto, Japan).

At this point of therapy we took a radiograph to allow subsequent execution of the following steps:

Glide path until to a full working length in both the buccal and palatal canals with a One G (300 rpm/5 Ncm) instrument.

Shaping canals in minimal invasive way was performed with TS1 (300 rpm/2 Ncm), 25/04, extending until to full length of the buccal canal, until to the merged point for the palatal canal in order to avoid an apical zip and hazardous stress with the endodontic instrument, especially dangerous when navigating second canal curvatures.

Apical gauging: the foramen was gauged introducing a 25/02 NiTi hand file, which was snug at its working length.

After the shaping procedure, in order to assure a three-dimensionally cleaning of the root canal system, an aqueous 17% solution of EDTA flooded into the pulp chamber was activated using a manual-dynamic activation by of some gutta-percha points for 120 seconds in each canal. After rinsing with physiological saline, a solution 6% of NaOCl flooded into the pulp chamber was activated again using a manual-dynamic activation for 120 seconds in each canal.

**Figure 1:**
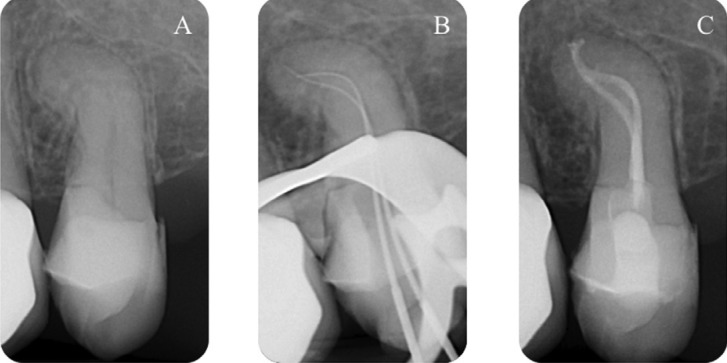
A) Pre-operative radiograph shows one root and an endodontic dilacerated anatomy, B) Peri-operative radiograph shows working length and endodontic anatomy, C) Post operative radiograph shows two well-obturated canals.

The canal system was then dried using sterile paper points. After having applied a drop of Kerr EWT pulp canal sealer (Kerr, Romulus, MI) with a coated paper point at the entrance of each canal, both canals were filled with Thermafil 2. The final radiographs showed two well-obturated canals of this single rooted maxillary premolar (Fig. 1).

Case 2

A 58-year-old caucasian female was referred to the Endodontics Clinic of the University of Strasbourg Dental Faculty for retreatment of a left maxillary second premolar. Endodontic treatment was performed in two sessions. Findings of a clinical examination revealed that the tooth had a prosthetic metal crown, that was tender on percussion with episodes of spontaneous pain.

The periapical radiographic examination, with different angle-shots, showed the presence of only one root with an unusual anatomic variation, and a probable endodontic dilacerated anatomy.

After local anesthesia and crown removal, the walls of the access cavity were reconstructed with SDR composite (DentsplySirona Ballaigues, Switzerland), thanks to the installation of an orthodontic band.

 A rubber dam was placed, the buccal and lingual canals were located with Start X1 using an operating microscope (Leika M320) and the previous perforation was visualized.

During the first session the perforation was treated with biodentine.

For this tooth we decided to plane using 2 different shaping techniques in order to compare the two different techniques.

During all instrumentation steps an aqueous 6% NaOCl solution was used for irrigation.

The lingual canal was treated with an usual technique:

manual scouting

glide path

shaping

In order to improve the access to the canal, a SX instrument (DentsplySirona Ballaigues, Switzerland),)(300 rpm/5 Ncm) from ProTaper Universal system was used.

 After the use of Sx opener a # 08 stainless steel K-file (DentsplySirona Ballaigues, Switzerland),) wasn’t able to go up to the WL, ), falling 8mm short of the WL.

In order to facilitate the apical scouting a # 10 K file was used short of 9 mm and # 15 K-file short of 10 mm.

So at the second wave # 08 K-file was 7mm short from the WL so again # 10 K file and # 15 K-file were used short of 8 and 9mm, respectively from the WL 

After having repeated the scouting sequence numerous times and extensive use of a pre-curved manual K file we ended the scouting step.

A mechanical glide-path with Proglider DentsplySirona Ballaigues, Switzerland), at working length was performed using an endodontic engine (300 rpm/5 Ncm).

Root canal preparation was performed using ProTaper Next X1(DentsplySirona Ballaigues, Switzerland) until to WL and ProTaper Next X2 (operating at 300 rpm and torque of 5 N/cm) until to 2.5 mm shorter from the WL. Then manual 20/02 and 25/02 NiTi hand file (Dentsply/Maillefer) sliding down the glide path up to working length, the shaping procedure finished after 58’ 45’’.

The buccal canal was treated with a modern step down-technique without initial manual scouting:

Initial preflaring above of second curve with in and out movement

Apical scouting

Glide path

Shaping

The initial mechanical preflaring was performed at first with proglider (Dentsplysirona) until to above the first root canal curvature applying an in and out movement, using an endodontic engine (300 rpm/5 Ncm).

Thanks to the initial preflaring using a #10 stainless steel K-file (Dentsply Maillefer) we scouted the canal up to working length + 0. 5 mm. Length determination was taken using an electronic apex locator (Root ZX; J Morita Co, Kyoto, Japan).

A mechanical glide-path with proglider at working length was performed using an endodontic engine (300 rpm/5 Ncm).

Root canal preparation was performed by preparing the root canals to working length with a ProTaper Next X1, a Pro Taper Next X2 (operating at 300 rpm and torque of 5 N/cm) 2.5 mm shorter from the WL. After manual 20/02 nd 25/02 NiTi hand file (Dentsply/Maillefer) sliding down the glide path up to working length. The shaping procedure was finished after 6’ 17’’.

After the shaping procedure, in order to assure a three-dimensionally cleaning of the root canal system, an aqueous 17% solution of EDTA was flooded into the pulp chamber was then activated using a manual-dynamic activation by a gutta-percha point for 120 seconds in each canal. After rinsing with physiological saline, a solution 6% of NaOCl flooded into the pulp chamber was activated using a manual-dynamic activation for 120 seconds in each canal.

**Figure 2:**
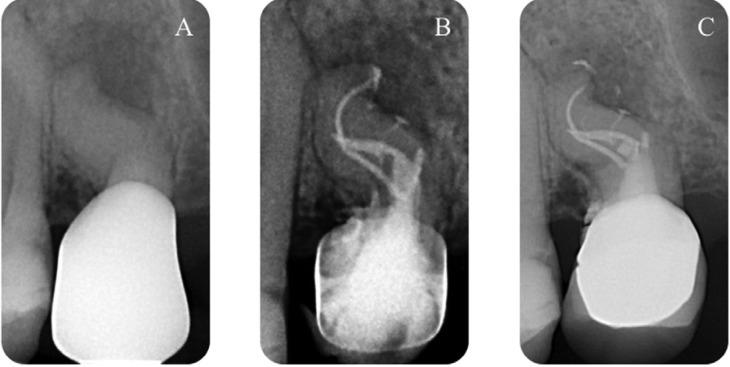
A) Pre-operative radiograph shows one root and an endodontic dilacerated anatomy, B) Post operative radiograph shows two well-obturated canals as well as the lateral canals, C) 1 year later, radiograph shows the healing of periapical lesion.

Then the canal system was dried using sterile paper points. After having applied a drop of EWT pulp canal sealer (Kerr) with a coated paper point in the entrance of each canal, both sites were filled with Thermafil 25. The final radiographs showed two well-obturated canals, with some lateral canal, of this single rooted maxillary premolar (Fig. 2).

## Discussion

Knowledge of the endodontic anatomy, its frequent variations and complexities, is essential for a successful endodontic treatment (1-4).

Maxillary 2nd premolars are usually considered as single-rooted teeth with a basic straight root canal system. As a matter of fact, according to data from *ex vivo* studies, two canals and more rarely, two dilacerated canals can occur. Clinicians frequently underestimate the importance of pre-operative radiographs and pre-access analysis before performing access cavity preparation and planning the correct shaping sequence.

In dilacerated anatomy, canal scouting step might cause some procedural errors such as ledge, apical zipping, canal straightening, elbow, blockage, fracture, leading to incomplete shaping of the root canal system, and to a possible failure of the root canal treatment. To avoid these procedural errors we propose a modern step down technique using at first a rotary NiTi glide path instrument, that goes up to the 2/3 of root canal length or until to the first impediment, in order to allow an easy apical scouting of the last millimeters of endodont and increase the volume of the irrigants in the apical region starting from the initial stages of the canal instrumentation.

Hence the 10 K file can work without any coronal interference, giving better control during the apical scouting, decreasing apical extrusion of debris, and reducing post-operative pain. After the preflaring step we can scouted the very difficult canals in easy way, reaching quickly into the apex and assessing the whole instrumentation steps as appearing faster and safer.

Maxillary premolars with dilacerated endodontic anatomy need a specific instrumental sequence in order to more quickly eliminate the interference at the coronal and middle tiers of the canal root system. To perform this step it is important to use a glide path rotary file with tip not larger than 16/100 and a taper not larger than 3% in the apical part.

In the preflaring step, the use of a classic opener, with deep taper and large tip, is less effective, considering that the opener is made to relocate and negotiate the first 2 mm of the root canal.

We propose a modern step-down technique able to transform a difficult canal into a normal canal, with an initial preflaring of the 2/3 of the root canal system, an apical scouting, usually with 10 K-file, glide path, and shaping.

Moreover this shaping protocol allows us to increase the volume of irrigants in the apical region starting from the initial stages of the canal instrumentation, and to avoid procedural errors leading to incomplete shaping of the root canal system, and to a possible failure of the root canal treatment.

It is clear that in the case of dilacerated endodontic anatomy we must reduce the taper of final shaping to avoid file separation and apical zip. Concerning the filling step, considering canal curvature and the small taper, in our opinion, the better technique would be to use a carrier based technique.
